# No QTc Prolongation in Girls and Women with Turner Syndrome

**DOI:** 10.1210/clinem/dgaa552

**Published:** 2020-08-24

**Authors:** Iris D Noordman, Anthonie L Duijnhouwer, Misty Coert, Melanie Bos, Marlies Kempers, Henri J L M Timmers, Zina Fejzic, Janiëlle A E M van der Velden, Livia Kapusta

**Affiliations:** 1 Department of Pediatric Endocrinology, Amalia Children’s Hospital, Radboud University Medical Center, Nijmegen, The Netherlands; 2 Department of Cardiology, Radboud University Medical Center, Nijmegen, The Netherlands; 3 Department of Neonatology, Willem Alexander Children’s Hospital, Leiden University Medical Center, Leiden, The Netherlands; 4 Department of Pediatric Cardiology, Amalia Children’s Hospital, Radboud University Medical Center, Nijmegen, The Netherlands; 5 Department of Human Genetics, Radboud University Medical Center, Nijmegen, The Netherlands; 6 Department of Internal Medicine, Radboud University Medical Center, Nijmegen, The Netherlands; 7 Pediatric Cardiology Unit, Dana-Dwek Children’s Hospital, Tel-Aviv Sourasky Medical Center, Sackler School of Medicine, Tel Aviv University, Tel Aviv, Israel

**Keywords:** Turner syndrome, electrocardiogram, QT interval, karyotype

## Abstract

**Context:**

Turner syndrome (TS) is a genetic condition that is reported to be associated with a prolonged rate-corrected QT (QTc) interval.

**Objectives:**

To evaluate the prevalence of QTc prolongation in patients with TS, to compare their QTc intervals with healthy controls, and to investigate whether QTc prolongation is associated with a monosomy 45,X karyotype.

**Method:**

Girls (n = 101) and women (n = 251) with TS visiting our center from 2004–2018 were included in this cross-sectional study. QT intervals of 12-leaded electrocardiograms were measured manually, using Bazett’s and Hodges formulas to correct for heart rate. A QTc interval of >450 ms for girls and >460 ms for women was considered prolonged. Corrected QT (QTc) intervals of patients with TS were compared to the QTc intervals of healthy girls and women from the same age groups derived from the literature.

**Results:**

In total, 5% of the population with TS had a prolonged QTc interval using Bazett’s formula and 0% using Hodges formula. Mean QTc intervals of these patients were not prolonged compared with the QTc interval of healthy individuals from the literature. Girls showed shorter mean QTc intervals compared with women. We found no association between monosomy 45,X and prolongation of the QTc interval.

**Conclusions:**

This study shows that the QTc interval in girls and women with TS is not prolonged compared with the general population derived from the literature, using both Bazett’s and Hodges formulas. Furthermore, girls show shorter QTc intervals compared with women, and a monosomy 45,X karyotype is not associated with QTc prolongation.

Turner syndrome (TS) is a chromosomal condition that affects phenotypic females who have 1 intact X chromosome and complete or partial absence of the second sex chromosome (1). Turner syndrome is associated with various cardiovascular abnormalities, such as a bicuspid aortic valve (BAV) and coarctation of the aorta (COA) (2). A number of studies have reported a higher prevalence of abnormal electrocardiogram (ECG) findings in small patient groups, of which prolongation of the corrected QT (QTc) interval was the most frequently suggested abnormality (3–9). A resting ECG at the time of diagnosis is therefore recommended (1). The mechanism behind QTc prolongation in TS is unknown, although there are theories suggesting that it is due to the loss of a sex chromosome or associated with the administration of sex hormones (10, 11).

In the general population, a prolonged QTc interval is associated with cardiac arrhythmia, torsades de pointes (a form of ventricular tachycardia), and even death (12). Whether the reported QTc prolongation in patients with TS contributes to a higher mortality rate in this population is not clear. The QTc interval can be calculated with both Bazett’s and Hodges formulas (13, 14). Studies describing QTc prolongation in small cohorts of girls and women with TS have mostly used Bazett’s formula. However, as suggested by the latest European Clinical Practice Guidelines for the care of girls and women with TS, Hodges formula is preferred, because of the described elevated intrinsic heart rate in this patient group (1, 4, 7, 8, 15, 16).

Evaluation of the QTc interval is challenging. The QT interval can be measured manually on printed or computerized ECGs, or automatically by a computer algorithm that measures the QT interval. The American Heart Association reported that it is essential to visually validate the QT interval reported by a computer algorithm, in view of the clinical importance of QTc interval prolongation (15). The definition of QTc prolongation is still under debate. Some studies suggesting QTc prolongation in TS used a threshold of 440 ms in girls and women, whereas the American Heart Association and other studies suggest a QTc threshold of 460 ms for women in general (15, 17, 18). For girls there is less evidence available, but 2 large studies suggested that a QTc interval >450 ms should be considered prolonged in children up to the age of 16 (19, 20).

Previous studies showed an association between karyotype and phenotype in patients with TS. Patients with a monosomy 45,X have a more severe phenotype compared to other patients, presenting with more dysmorphic features, cardiovascular abnormalities, and other comorbidities (21, 22). The association between karyotype and QTc prolongation was rarely studied, and the outcomes were contradicting (3, 5, 8).

Our objectives were (1) to evaluate the prevalence of QTc prolongation with both Bazett’s and Hodges formulas, (2) to compare the mean QTc intervals of girls and women with TS to those of healthy age-matched references derived from the literature, and (3) to evaluate whether QTc prolongation is associated with a monosomy 45,X karyotype in a large population of patients of all ages with TS.

## Methods

### Patients

In this cross-sectional study, we included all patients with clinically and genetically diagnosed TS, who visited the Turner Center of the Amalia Children’s Hospital and Radboud University Medical Center between 2004 and 2018. The printed ECGs of girls (1–16 years old) and the computerized ECGs of women (>16 years old) were evaluated. We chose these age categories because most studies on normal QTc values in children included patients younger than 17 years old. Children <1 year were not included, because the cutoff value in this patient group is under debate. This study was approved by the local ethics committee.

Data on age, karyotype, hypertension, growth hormone treatment, and cardiac malformations were obtained from the medical records. The use of possible QTc-prolonging drugs was reviewed using the CredibleMeds list (23). Hypertension was defined as the need for antihypertensive medication to normalize the blood pressure. The presence or absence of cardiac malformations was diagnosed by echocardiography in children, and by echocardiography and magnetic resonance imaging in adults according to the (inter)national protocol (1). Information about mortality was obtained consulting the Municipal Personal Records Database.

All karyotypes, analyzed under supervision of a clinical geneticist (M.K.), were classified into 7 subgroups: monosomy 45,X; mosaicism 45,X/46,XX; isochromosome (eg, 46,X iso (X), 45,X/46,X iso (X)); deletion (eg, 46,X del (X), 45,X/46,X del (X)); multiple cell lines (eg, 45,X/47,XXX, 45,X/46,XX/47,XXX); ring X (eg, 46,X ring (X), 45,X/46,X ring (X)); and Y-material (eg, 45,X/46,XY). If it was not possible to classify a karyotype into 1 of the karyotype groups (eg, if karyotype information was available but not complete), it was categorized as “nonclassifiable” and excluded from karyotype analysis (n = 3). The karyotype was examined in lymphocytes (30 cells) and/or buccal cells (100 cells). Since monosomy of the X chromosome (45,X) can be more reliably established in buccal cells than in a blood sample, the karyotype of the buccal cells was used for classification when available (24). To search for associations between monosomy 45,X and QTc prolongation, the monosomy 45,X group was compared with patients with other karyotypes.

### Electrocardiogram

Twelve-leaded ECGs were recorded at 25 mm/second, with an amplitude of 10 mm/mV. The QT interval was measured manually on the printed or computerized ECGs from the onset of the QRS complex to the end of the T-wave. The QT interval was preferably measured 3 times in lead II. The teach-the-tangent method was used to exclude the U-wave (15). The correction of the QT interval was done with both Bazett’s (QTc = QT / √ RR [previous RR interval]) and Hodges formula (QTc = QT + 1.75 [heart rate - 60]) (13, 14). Use of both formulas ensures that our study could be compared with prior studies and that the Hodges formula will eliminate the strong correlation with heart rate. Measurements were carried out by 2 researchers (M.B., I.N.) under supervision of 2 pediatric cardiologists (Z.F., L.K.). Both researchers were blinded to the karyotype and the medical history of the patients.

A QTc interval >450 ms was considered prolonged for girls until the age of 16 years old (19, 20). For women, QTc prolongation was defined as a QTc interval >460 ms (15). Based on these definitions, the prevalence of QTc prolongation using both Bazett’s and Hodges formula was evaluated and compared with other studies investigating the prevalence of QTc prolongation in patients with TS. As some of these studies defined QTc prolongation as a QTc interval >440 ms, we also evaluated the prevalence of QTc intervals using this specific time interval definition. A QTc interval of <340 ms was defined as a short QTc interval for both girls and women (25, 26).

To investigate the possible difference between the QTc interval of patients with TS and the general population, we performed a literature search. We selected studies with a large number of healthy girls and/or women (n > 700) that were divided into age groups and that reported the mean/median QTc interval using both Bazett’s and Hodges formulas. We created the same age groups in our TS cohort and compared the QTc intervals of our patients to those derived from the age-matched healthy cohort (Table 3) (27–29).

### Statistics

Statistical analyses were performed with SPSS version 25.0. Descriptive statistics were used to describe baseline characteristics of the study population. The normality of continuous variables was determined using visual and statistical methods (eg, shape of histogram, skewness, and kurtosis). Values were given as mean ± standard deviation (SD) or median (min–max), depending on the normality of the variables. The QTc interval of girls/women with TS was compared to the QTc interval of healthy individuals from the same age group (derived from the literature), using 1-sample *t*-tests. Unpaired *t*-tests or Mann-Whitney U tests and Chi-square tests were performed to compare the QTc interval between girls and women within our population with TS, and to compare the 45,X group with the “other karyotype” group. A *P*-value of <0.05 was considered statistically significant.

Inter- and intraobserver reliability were expressed as intraclass correlation coefficients (ICCs). Intraclass correlation coefficient estimates and their 95% confidence intervals (CIs) were calculated based on an absolute agreement, single measurement, 2-way mixed-effects model. An ICC <0.20 was considered as poor agreement, 0.21–0.40 as fair agreement, 0.41–0.60 as moderate agreement, 0.61–0.80 as good agreement, and 0.81–1.00 as very good agreement (30). We decided to consider an agreement of >0.80 as acceptable for the inter- and intraobserver reliability in this study.

## Results

In total, 426 patients (120 girls and 306 women) were eligible for this study. We excluded 74 patients because of age <1 year (n = 4), no karyotype available or other genetic conditions associated with cardiac malformations (n = 19), no informed consent (n = 11), no ECG available or poor quality (n = 39), and the presence of a male phenotype (n = 1). After exclusion, the total study population consisted of 352 patients with TS (101 girls and 251 women).

Demographic and clinical characteristics are presented in Table 1. The median age of the total population was 23 years (1–65 years), 11 years (1–16 years) for girls, and 30 years (17–65 years) for women. The most common karyotype in our population was monosomy 45,X, followed by isochromosome and mosaicism 45,X/46,XX.

**Table 1.** The Demographic and Clinical Characteristics of the Total Cohort, Girls, and Women with Turner Syndrome

	Total Cohort (n = 352)	Girls (n = 101)	Women (n = 251)
Age, years; median (min–max)	23 (1–65)	11 (1–16)	30 (17–65)
Hypertension (n, %)	46 (13)	4 (4)	42 (17)
BAV (n, %)	76 (22)	21 (21)	55 (22)
COA (n, %)	18 (5)	10 (10)	8 (3)
GH^*a*^ (n, %)	233 (66)	80 (79)	153 (61)
**Karyotype (n, %)**			
Monosomy 45,X	120 (34.1)	33 (32.7)	87 (34.7)
Mosaicism 45,X/46,XX	64 (18.1)	24 (23.8)	40 (15.9)
Isochromosome	72 (20.5)	18 (17.8)	54 (21.5)
Deletion	31 (8.8)	9 (8.9)	22 (8.8)
Multiple cell lines	21 (6.0)	7 (6.9)	14 (5.6)
Ring	18 (5.1)	4 (4.0)	14 (5.6)
Y-material	23 (6.5)	5 (5.0)	18 (7.2)
Not to classify	3 (0.9)	1 (1.0)	2 (0.8)

Values are expressed as median (min–max) and n (%). In 2 cases, information on hypertension was missing.

Abbreviations: BAV, bicuspid aortic valve; COA, coarctation of the aorta; GH, growth hormone; max, maximum; min, minimum.^*a*^Currently treated with GH or treated with GH in the past.

The mean heart rate, QT-interval, and QTc interval of the total cohort, girls, and women with TS are shown in Table 2. The mean QTc interval of the total population using Bazett’s formula was longer compared with Hodges formula (420 and 400 ms [*P* < 0.001], respectively). Girls with TS had a shorter QTc interval compared with women with TS, regardless of the correction formula. Of all patients, 5% (n = 19) had a prolonged QTc interval according to Bazett’s and 0% (n = 0) according to Hodges formula. When using a threshold of 440 ms, 18 girls (18%) and 55 women (22%) had a prolonged QTc interval using Bazett’s formula. With Hodges formula, no girls and 11 women (4%) had a QTc interval >440 ms. No patients, either girls or women, had a Bazett or Hodge QTc interval longer than 500 ms. Furthermore, no patients showed a short QTc interval of <340 ms.

**Table 2.** The Mean Heart Rate, QT Interval, and QTc Interval of the Total Cohort, Girls, and Women with Turner Syndrome

		Total Cohort (n = 352)	Girls (n = 101)	Women (n = 251)	*P*-value
Heart rate	bpm	85 ± 18	95 ± 18	81 ± 16	<0.001
QT interval	ms	356 ± 33	332 ± 30	366 ± 30	<0.001
QTc Bazett	ms	420 ± 25	414 ± 25	422 ± 25	0.011
Prolonged bQTc	n (%)	19 (5)	4 (4)	15 (6)	0.449
QTc Hodges	ms	400 ± 19	392 ± 19	403 ± 19	<0.001
Prolonged hQTc	n (%)	0 (0)	0 (0)	0 (0)	*–*

Values are expressed as mean ± SD. Differences between girls and women were tested with *t*-tests and Chi-square tests.

Abbreviations: bpm, beats per minute; bQTc, QTc interval with Bazett’s formula; hQTc, QTc interval with Hodges formula; ms, millisecond; QTc, corrected QT interval.

Table 3 shows the QTc intervals of girls and women with TS divided into age groups, compared with the QTc intervals of healthy girls and women from the same age groups, as derived from the literature. Girls and women with TS show comparable, or even shorter QTc intervals, compared with the reference group.

**Table 3.** QTc Intervals in Patients with Turner Syndrome Versus QTc of Healthy Girls and Women from the Literature

	Patients with TS			Reference Population				
	**TS Girls**			**Qiu 2007 (27)**				
	**N**	**bQTc**	**hQTc**	**N**	**bQTc**	**hQTc**	**P-bQTc**	**P-hQTc**
**1–5 y**	11	410 ± 23	394 ± 21	292	415	395	0.480	0.841
**6–10 y**	39	416 ± 25	391 ± 18	288	418	399	0.698	0.008
**11–15 y**	39	413 ± 25	393 ± 19	341	419	405	0.149	<0.001
	**TS Women**			**Rijnbeek 2014 (28) **				
	**N**	**bQTc**	**hQTc**	**N**	**bQTc**	**hQTc**	**P-bQTc**	**P-hQTc**
**16–19 y**	50	418 ± 22	397 ± 18	385	429	414	<0.001	<0.001
**20–29 y**	87	419 ± 25	399 ± 17	868	418	409	0.808	<0.001
**30–39 y**	61	426 ± 25	408 ± 20	1435	419	412	0.024	0.152
**40–49 y**	45	424 ± 26	406 ± 19	597	421	412	0.465	0.029
**50–59 y**	17	423 ± 31	405 ± 20	1077	427	416	0.612	0.034
**60–69 y**	3	421 ± 18	398 ± 14	1124	429	416	0.513	0.161
	**TS Girls**			**Palhares 2017 (29) **				
	**N**	**bQTc**	**hQTc**	**N**	**bQTc**	**hQTc**	**P-bQTc**	**P-hQTc**
**1–2 y**	2	438 ± 6	430 ± 11	216	421	404	0.170	0.179
**3–4 y**	6	398 ± 18	383 ± 13	667	424	401	0.014	0.017
**5–7 y**	14	415 ± 21	388 ± 11	1947	425	403	0.109	<0.001
**8–11 y**	38	415 ± 26	392 ± 19	4601	427	407	0.006	<0.001
**12–15 y**	29	415 ± 26	394 ± 20	10709	426	409	0.025	0.001
	**TS Women**			**Palhares 2017 (29) **				
	**N**	**bQTc**	**hQTc**	**N**	**bQTc**	**hQTc**	**P-bQTc**	**P-hQTc**
**16–19 y**	50	418 ± 22	397 ± 18	14453	423	408	0.144	<0.001
**20–29 y**	87	419 ± 25	399 ± 17	43281	426	411	0.008	<0.001
**30–39 y**	61	426 ± 25	408 ± 20	55671	429	414	0.432	0.030
**40–49 y**	45	424 ± 26	406 ± 19	57942	432	418	0.045	<0.001
**50–59 y**	17	423 ± 31	405 ± 20	46563	435	421	0.132	0.004
**60–69 y**	3	421 ± 18	398 ± 14	28620	437	423	0.260	0.093

Values given are mean ± SD (in ms) for patients with TS, and mean or median in ms for the reference groups (27–29). The QTc interval of girls/women with TS was compared with the QTc interval of healthy individuals from the same age group, derived from the literature, with a 1-sample *t*-test (P-bQTc/P-hQTc). The study of Palhares et al (29) included both children and adults, so this study was used as a reference for both girls and women with TS. Methods may differ between the different studies.

Abbreviations: bQTc, QTc interval with Bazett’s formula; hQTc, QTc interval with Hodges formula; P-bQTc, P-value calculated for the QTc interval with Bazett's formula; P-hQTc, P-value calculated for the QTc interval with Hodges formula; QTc, corrected QT interval; TS, Turner syndrome; y, year.

### Cardiac malformations and hypertension

The presence of BAV (22% of the population, see Table 1) in girls and women with TS did not result in a statistically significant longer QTc interval using Bazett’s (420 ± 28 ms vs 420 ± 25 ms, *P* = 0.945) and Hodges (401 ± 21 ms vs 400 ± 19 ms, *P* = 0.462) formulas. Also, the presence of COA (5% of the population, see Table 1) in girls and women with TS did not result in a statistically significant longer QTc interval using both formulas. Patients with hypertension (13% of the population, see Table 1) showed statistically significant longer QTc intervals with Bazett’s formula (430 ± 28 ms vs 418 ± 24 ms; *P* = 0.002) and Hodges formula (410 ± 19 ms vs 398 ± 19 ms; *P* < 0.001), compared with patients without hypertension. However, most of the patients with hypertension were women (4 girls and 42 women).

### Karyotype: monosomy 45,X versus “other karyotype”

The karyotype and relevant demographic and clinical data are shown in Table 4. In addition to blood, 36% of the karyotypes of the total population were determined in a second cell line (buccal cells); of the 120 subjects with monosomy 45,X, 69% of the karyotypes were determined in both blood and buccal cells. In 25 patients of the total population (7%), the blood originally showed a 45,X karyotype, whereas the buccal cells displayed another karyotype.

**Table 4.** The Basic Characteristics of the Monosomy 45,X Group and the “Other Karyotype” Group

	45,X (n = 120)	Other Karyotype (n = 229)	*P*-value
Age (years)	23 (2–65)	24 (1–61)	0.570
Hypertension (n, %)	16 (13)	29 (13)	0.883
BAV (n, %)	34 (28)	40 (17)	0.018
COA (n, %)	9 (8)	9 (4)	0.152
GH (n, %)	88 (73)	142 (62)	0.034

Values are expressed as median (min-max). Differences between the 45,X and “other karyotype” group were tested with Mann-Whitney U tests and Chi-square tests. Three patients with a “not to classify” karyotype were excluded from karyotype analysis.

Abbreviation: BAV, bicuspid aortic valve; COA, coarctation of the aorta; GH, growth hormone; max, maximum; min, minimum.

Both karyotype groups were similar in terms of age and hypertension. The presence of a BAV was more common in the 45,X group compared with the “other karyotype” group (28% vs 17%, respectively), and more patients in the 45,X karyotype group were treated with growth hormone (73% vs 62%, respectively). Table 5 shows that patients with a monosomy 45,X karyotype had a higher basic heart rate compared with the patients with other karyotypes (89 ± 18 ms vs 83 ± 17 ms; *P* = 0.002). There was no statistically significant difference between QTc interval of patients with monosomy 45,X compared with patients with other karyotypes, when using both formulas.

**Table 5.** The Mean Heart Rate, QT Interval, and QTc Interval of the Total Cohort, Monosomy 45,X Group, and the “Other Karyotype” Group in Turner Syndrome

		Total Cohort (n = 349^*a*^)	45,X (n = 120)	Other Karyotype (n = 229)	*P*-value
Heart rate	bpm	85 ± 18	89 ± 18	83 ± 17	0.002
QT interval	ms	356 ± 33	351 ± 35	358 ± 33	0.056
QTc Bazett	ms	420 ± 25	423 ± 26	418 ± 24	0.060
Prolonged bQTc	n (%)	18 (5)	7 (6)	11 (5)	0.679
QTc Hodges	ms	400 ± 19	402 ± 19	398 ± 19	0.084
Prolonged hQTc	n (%)	0 (0)	0 (0)	0 (0)	–

Values are expressed as mean ± SD. Differences between the 45,X and “other karyotype” group were tested with *t*-tests and Chi-square tests.

Abbreviations: bpm, beats per minute; bQTc, QTc interval with Bazett’s formula; hQTc, QTc interval with Hodges formula; ms, millisecond; QTc, corrected QT interval.

^*a*^Three patients with a “not to classify” karyotype were excluded from karyotype analysis.

### Growth hormone

The use of growth hormone was not associated with a longer QTc interval when using Bazett’s formula (419 ± 25 vs 422 ± 26 ms, *P* = 0.336). It even showed a statistically significant shorter QTc interval when using Hodges formula (398 ± 18 ms vs 403 ± 20 ms, *P* = 0.02). Furthermore, the heart rate in the growth hormone group was higher compared with patients that did not use growth hormone (87 ± 17 vs 82 ± 18 bpm (*P* = 0.025)).

### QTc prolonging medication

From the total population, 4 patients (1%) used QTc-prolonging medication at the time of the ECG, according to the CredibleMeds list ((es)citalopram [n = 3] and domperidone [n = 1]). None of these patients had a prolonged QTc interval using both correction formulas (402, 416, 442, and 454 ms using Bazett’s formula, and 395, 430, 440, and 445 ms using Hodges formula).

### Mortality

In the total Turner population, 5 patients died at age 44–67 years. Causes of death were aortic dissection (n = 2), intestinal ischemia (n = 1), non-Hodgkin lymphoma (n = 1), and unknown (n = 1). None of these patients had a QTc interval of >460 ms at the time of the ECG (Bazett: 401–434 ms; Hodge: 383–412 ms).

### Inter- and intraobserver variability

Inter- and intraobserver variability in this study was very low. Agreement between the 2 researchers measuring QT intervals was good, with an ICC of 0.90 (95% CI, 0.76–0.96). Intraobserver reliability was very good with an ICC of 0.97 (95% CI, 0.92–0.99).

## Discussion

This is a unique study investigating the QTc interval in a very large population of 352 girls and women with TS, using both Bazett’s and Hodges formulas for the correction of the QT interval. We show no QTc prolongation using Hodges formula in girls and women with TS. Mean QTc intervals are not prolonged when compared with the QTc intervals of healthy individuals of the same age group derived from the literature. Girls with TS show significantly shorter QTc intervals compared with women with TS, and there is no difference in QTc prolongation between monosomy 45,X karyotype and other karyotypes. This suggests that QTc prolongation is not a specific feature of girls and women with TS.

We found a clear difference in the prevalence of QTc prolongation using Bazett’s and Hodges formulas, since only Hodges formula (linear approach) adequately corrects for the high heart rate in patients with TS (1, 8, 15). This current study describes a much lower prevalence of QTc prolongation compared with other recent studies investigating the QTc interval in patients with TS (Bazett: 11–36%, Hodges 7–15%) (3–5, 8). It is important to note the differences between our study and previous studies investigating the QTc interval in patients with TS, which could explain the differences in the results. At first, in our study, different cutoff values were used for the definition of QTc prolongation (450 ms for girls and 460 ms for women, compared with 440 ms in some other studies) (15, 17–20). When we used 440 ms as a cutoff value for girls and women with TS, we showed prolonged QTc intervals with Bazett’s formula of 18% and 22% in girls and women, respectively. With Hodges formula, we found 0% and 4% QTc prolongation using this cutoff value. These results show that our findings, when using Bazett’s formula, are comparable to previous studies, but we have interpreted the results differently according to the American Heart Association and other studies advising for cutoff values and the use of Hodges formula, as mentioned before.

We compared our QTc results to the general population, as derived from literature, and showed that the QTc interval in girls and women from different age groups was not prolonged. Mean QTc intervals using both formulas were even shorter compared with the general population in some age groups, although this difference seems to be of minor clinical relevance.

We found no statistically significant difference between the QTc interval of patients with karyotype 45,X and other karyotypes. Previous studies showed conflicting results in small study populations. Bondy et al did not find an association between karyotype and QTc prolongation in girls with TS, although the number of individuals with a normal cell line in this study was small (3). On the contrary, Dalla Pozza et al and Trolle et al did find a longer QTc interval (with Bazett’s and Hodges formulas) in patients with karyotype 45,X compared with other karyotypes, with mean differences of 10 ms (*P* < 0.05) and 10.6 ms (*P* = 0.055), respectively (5, 8). However, it is questionable whether this difference is clinically relevant. The percentage of patients with karyotypes other than monosomy 45,X was high in our population. This is probably because we included buccal cell analysis to determine karyotypes, and not only lymphocytes, which changed the karyotype in 7% of the patients with an original monosomy 45,X karyotype. This suggests that, in case a monosomy 45,X karyotype is diagnosed in lymphocytes, a buccal sampling should be considered.

In this study, we showed that TS patients with hypertension have longer QTc intervals compared to patients without hypertension. Previous research has shown the same association between hypertension and prolonged QTc interval in the general population (31, 32). Thus, the prevalence of QTc prolongation in patients with TS and hypertension was still comparable to the general population (with hypertension). Also, the presence of BAV or COA was not associated with longer QTc intervals in our cohort. The use of growth hormone was associated with a shorter QTc interval using Hodges formula, although the difference was only 5 ms. This association might be explained by the fact that more girls used growth hormone compared with women, and girls show shorter QTc intervals compared with women.

As reported by Yap et al, the most common cause of acquired long QT syndrome is drug-induced (12). None of the patients in our population using QT-prolonging drugs showed a prolonged QTc interval. Furthermore, none of our patients used antiarrhythmics, which are described as the most common cause of acquired long QT syndrome.

A prolonged QTc interval >500 ms was not observed in our study. This is important, because previous studies showed that a QTc interval of >500 ms is associated with sudden cardiac death and torsades de pointes, which means these patients are possibly more at risk (33). Patel et al reported in a large study in patients with sinus tachycardia (baseline sinus rate of 100 bpm), that a prolonged Bazett QTc but normal Hodges QTc did not have a significantly increased risk of cardiovascular events when compared with the normal Bazett and Hodge QTc (34). Studies investigating QTc prolongation in patients with TS showed no association with sudden cardiac death.

Our study has some limitations. We measured the QT interval manually, which means it may be difficult to compare our results with studies measuring the QT interval with a computer algorithm. It is suggested that the automatically measured QT interval is often longer than the QT interval as measured in any individual lead (15). Furthermore, we did not study a healthy population as a control group, but we chose to compare our results to international, well-accepted references (data derived from the literature). According to clinical practice, the previous RR interval of the QT interval was used for Bazett’s formula. For Hodges formula, heart rate was used, which might result in different values.

## Conclusion and Recommendations

This large study, including 352 girls and women with TS, shows that QTc prolongation is not a special feature of girls and women with TS. Even in patients with BAV, COA, and monosomy 45,X, we found no statistically significant longer QTc interval compared to other patients with TS. Only patients with hypertension showed a prolonged QTc interval, as is described in the general population.

In the international TS guideline, the presence of QTc prolongation in TS was the main reason for the recommendation of performing an ECG at diagnosis (1). Since we show no QTc interval prolongation using Hodges formula and mean QTc intervals comparable to healthy individuals derived from the literature, we question whether ECG should be a routine examination for this indication in patients with TS without other clinical indications for it. Furthermore, ECG follow up of TS patients with and without prolonged QTc intervals should not differ from the guidelines of the general population.

## Data Availability

The datasets generated during and/or analyzed during the current study are not publicly available but are available from the corresponding author on reasonable request.

## Additional Information

***Disclosure Summary:*** The authors have nothing to disclose.
